# Material Use for Air Leakage Prevention Post-Lung Resection: Insights from Real-World Data in Japan

**DOI:** 10.5761/atcs.oa.25-00235

**Published:** 2026-03-31

**Authors:** Hiraku Kumamaru, Shiori Nishimura, Hiroaki Miyata, Paola Mussi, Kazunobu Miyoshi, Akihiro Maki, Yugo Tanaka

**Affiliations:** 1 Department of Healthcare Quality Assessment, The University of Tokyo Graduate School of Medicine, Tokyo, Japan; 2 Department of Health Data Science, Yokohama City University Graduate School of Data Science, Yokohama, Kanagawa, Japan; 3 Johnson and Johnson K.K. Medical Company, Tokyo, Japan; 4 Division of General Thoracic Surgery and Breast and Endocrine Surgery, Faculty of Medicine, Tottori University, Yonago, Tottori, Japan

**Keywords:** materials, prolonged leakage, utilization, cost

## Abstract

**Purpose:**

Lung resection is a standard treatment for localized lung cancer, but prolonged air leakage is a common complication with health and economic burdens. Interventions such as staplers, sealants, and adjunctive materials are used, yet real-world data describing their utilization remain limited.

**Methods:**

Using a hospital database with claims and discharge summaries from 393 hospitals in Japan, we identified patients undergoing lobectomy, segmentectomy, or partial resection for primary lung cancer between 2015 and 2020. We evaluated intraoperative use of staple-line buttresses, fibrin glue, polyglycolic acid (PGA) sheets, and other materials. Patient characteristics and comorbidities were compared across material-use categories, and the incidence of prolonged postoperative drainage (≥7 days) was assessed.

**Results:**

Among 33094 lobectomy, 5443 segmentectomy, and 8242 partial resection patients, buttress use was uncommon (2.2%, 3.5%, and 6.4%). Patients receiving buttresses or PGA plus fibrin glue had higher prevalences of emphysema and interstitial pneumonia than those in the stapler-only group. Prolonged drainage remained frequent (6.9% in stapler-only vs. 24.8% in buttress among lobectomy patients) and was associated with nearly doubled postoperative in-hospital costs.

**Conclusions:**

Prolonged air leakage remains a major complication following lung resection, associated with clinical and economic burden. This study provided a nationwide overview of real-world utilization patterns of adjunctive materials.

## Introduction

Lung resection remains a standard treatment option for complete cure of localized lung cancer.^1,2)^ Prolonged air leakage is one of the most common complications after lung resection that can lead to health and economic burdens.^3)^ Numerous perioperative management techniques and medications, as well as medical devices and materials, have been developed to prevent air leakage after lung resection.^4–6)^ These include surgical staplers, staple-line buttresses,^7,8)^ topical sealants,^9–11)^ and biological sealants.^12–14)^ While these materials are available, there is a lack of comprehensive documentation regarding their real-world use in routine clinical practice after lung resection.

This study aimed to describe the real-world use of medical devices and materials to prevent prolonged air leakage after lung resection for primary lung cancer in Japan using a large administrative hospital-based database. It should be noted that it was not designed to evaluate the preventive effectiveness of these materials. The available hospital administrative data were insufficient to adequately adjust for differences in patient characteristics across the material-use groups. We also evaluated the incidence of prolonged air leakage after lung resection, using the extended drainage period accurately documented in the administrative database as an indicator for possible air leakage development. This study provides a nationwide overview of routine clinical practice and highlights real-world patterns of material utilization and associated outcomes that are not captured in controlled trial settings.

## Materials and Methods

### Data source

We utilized the Diagnosis Procedure Combination (DPC) database provided by the Medical Data Vision Co. Ltd. (MDV, Tokyo, Japan), comprising DPC data from 393 hospitals throughout Japan. The database covers 22% of all hospitals that employ the DPC payment system.^15)^ The DPC database is a patient-level medical claims database with additional clinical data recorded for each hospitalization episode, including cancer staging and patients’ smoking history.^16)^ The study was reviewed and approved by the ethics committee of the University of Tokyo Graduate School of Medicine (2021229NI).

### Study population

We identified patients who had undergone open or thoracoscopic lobectomy, segmentectomy, or partial lung resection between April 2015 and August 2020. Patients under the age of 20, those who had pneumonectomy, or underwent bronchoplasty or carinal resection/construction were excluded. Additionally, we excluded patients with unidentifiable resected lobe, those who underwent combined resection of neighboring organs, those involved in robotic-assisted procedures, and those without the use of staplers.

### Exposure: intraoperative material use categorization

Material utilization was ascertained from billing records for surgical staplers, staple-line buttresses, polyglycolic acid sheets (PGAS), fibrin glue, and other hemostatic/sealing agents (full list in **Supplementary Table 1**).

To improve interpretability, procedures were classified by the functional reinforcement strategy applied to the staple line. Categories were mutually exclusive and assigned as below, with the presence of a buttress taking precedence over other materials.

**Stapler-only group:** Stapler use with no adjunctive reinforcement or sealant.

**Buttress group:** Stapler used with a staple-line buttress, with or without additional sealants or other adjuncts.

**PGAS/Sealant group:** Stapler with PGAS and/or fibrin glue only, with no buttress and no other adjunctive sealing materials.

**PGAS/Sealant + Hemostatics group:** Stapler with PGAS and/or fibrin glue together with other non-buttress sealing materials.

**Other materials group:** Stapler with adjunctive materials not fitting Groups A–D (e.g., non-PGAS/non-fibrin sealants without buttress).

This classification was developed considering the presumed usage of materials corresponding to the potential risk of patients developing postoperative air leakage. We hypothesized that patients in the Buttress group would have the highest risk of postoperative air leakage, followed by those in the PGAS/Sealant + Hemostatics group. Those in the PGAS/Sealant group would have a moderate risk, and those in the Stapler-only group and the Other materials group would be at the lowest risk among the 5 groups.

### Patient and hospital characteristics

Information such as age, sex, initial diagnoses, comorbidities at admission, complications during hospitalization, body mass index (BMI), smoking history, and lung cancer TNM stage were extracted from the discharge summary data within the DPC database. In addition, we extracted data on whether the hospitals were designated as regional cancer treatment centers and on hospital size quantified by the number of beds from the database.

### Outcomes

Our primary outcome of interest was the occurrence of prolonged drainage, which we defined as the placement of drainage for 7 or more days following surgery. Additionally, we assessed the incidence of drainage placement for 3 or more days, as well as reoperation due to lung fistula and the utilization of adhesion therapy drugs on or after the first postoperative day.

We also evaluated the length of hospital stay and the associated hospitalization costs from the day following the surgical procedure until the patient’s discharge. We categorized the costs into the following components: bed and facility fees, surgery and intervention, laboratory examination and pathology, diagnostic imaging, nonsurgical procedures, medications, injection/intravenous fluid, and anesthesia.

### Statistical analysis

We summarized the characteristics of both patients and hospitals according to the intraoperative material-use combination group and the procedures performed. We also evaluated the incidences of the outcomes and the postoperative lengths of stay across the different material-use groups. In-hospital costs were evaluated based on the presence of prolonged drainage lasting <7 days or longer. No adjustment for hospital or patient characteristics was performed when comparing outcome incidences across material-use groups, as evaluating the effectiveness of the materials was not the objective of this study.

## Results

**Fig. 1:**
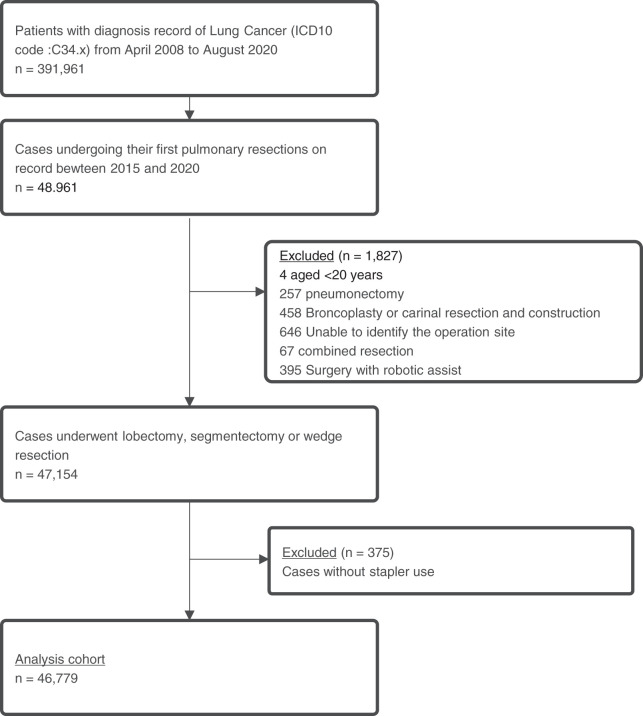
Cohort flow diagram

We identified a total of 46779 patients with a diagnosis of primary lung cancer who underwent pulmonary resection (**Fig. 1**). Among them, 33094 patients (70.7%) underwent lobectomy, 5443 patients (11.6%) underwent segmentectomy, and 8242 patients (17.6%) underwent partial resection (**Table 1**). Among the patients who underwent lobectomy, 7251 (21.9%) were in the Stapler-only group, while 743 (2.2%) were in the Buttress group. The PGAS/Sealant group included 14329 (43.3%) patients, and the PGAS/Sealant + Hemostatics group included 6228 (18.8%). Among the 5443 patients who underwent segmentectomy, 1021 (18.8%) were in the Stapler-only group, 190 (3.5%) were in the Buttress group, 2958 (54.3%) were in the PGAS/Sealant group, and 748 (13.7%) were in the PGAS/Sealant + Hemostatics group. For the 8242 patients who underwent partial resection, the proportion in the Buttress group was the highest among the 3 pulmonary resection categories, at 531 (6.4%). Partial resection patients also had the highest proportion in the Stapler-only group at 4446 (53.9%). In contrast, the PGAS/Sealant + Hemostatics group represented the lowest proportion among patients undergoing partial resection at 402 (4.9%).

**Table 1:** Use of medical materials by resection type for 46779 patients undergoing lung resection between April 2015 and August 2020

	Stapler plus				
	A) Stapler only	B) Buttress	C) PGAS and/or fibrin glue	D) PGAS and/or fibrin glue + others	E) Others
Lobectomy (n = 33094 (70.7%))					
N (%)	7251 (21.9)	743 (2.2)	14329 (43.3)	6228 (18.8)	4543 (13.7)
Segmentectomy (n = 5443 (11.6%))					
N (%)	1021 (18.8)	190 (3.5)	2958 (54.3)	748 (13.7)	526 (9.7)
Partial resection (n = 8242 (17.6%))					
N (%)	4446 (53.9)	531 (6.4)	2298 (27.9)	402 (4.9)	565 (6.9)

PGAS: polyglycolic acid sheets

### Patient characteristics associated with material use

The median age of all sampled patients was 71 years, and 60.2% were male. Among the lobectomy patients, the median age showed similar values across the 5 material-use groups (**Table 2**). The highest frequency of emphysema/chronic obstructive pulmonary disease (COPD) diagnosis in patients was observed in the Buttress group (40.8%), followed by similar frequencies in the PGAS/Sealant group (18.8%) and the PGAS/Sealant + Hemostatics group (19.5%), and the lowest frequency was found in the Stapler-only group (9.3%). The proportions of males, patients with an interstitial pneumonia (IP) diagnosis, those with low BMI, and individuals with a smoking history of pack-years ≥30 were higher in the Buttress group compared to other groups. However, the variability between the other groups was small. The Buttress group had the highest proportion of patients with larger tumors (T2 and above) and more lymph node metastasis (N1 and above) (**Supplementary Table 2**). However, the differences in tumor size and lymph node metastasis levels across the material-use groups were generally limited.

**Table 2:** Patient characteristics by material use combination and procedure type

	Stapler plus				
	A) Stapler only	B) Buttress	C) PGAS and/or fibrin glue	D) PGAS and/or fibrin glue + others	E) Others
Lobectomy (n = 33094)					
N	7251	743	14329	6228	4543
Median age (IQR) (years)	70.0 (64.0, 75.0)	72.0 (67.0, 77.0)	71.0 (65.0, 76.0)	71.0 (65.0, 76.0)	71.0 (65.0, 76.0)
Men, n (%)	3646 (50.3)	591 (79.5)	9063 (63.3)	4082 (65.5)	2664 (58.6)
Emphysema/COPD, n (%)	672 (9.3)	303 (40.8)	2693 (18.8)	1212 (19.5)	633 (13.9)
Interstitial pneumonia, n (%)	223 (3.1)	48 (6.5)	537 (3.8)	254 (4.1)	199 (4.4)
BMI <18 kg/m^2^, n (%)	357 (5.0)	58 (7.9)	838 (5.9)	393 (6.4)	240 (5.3)
Pack-years ≥30, n (%)	2318 (33.6)	397 (57.2)	5747 (42.8)	2507 (44.9)	1666 (41.5)
Segmentectomy (n = 5443)					
N	1021	190	2958	748	526
Median age (IQR) (years)	71.0 (65.0, 76.0)	74.0 (69.0, 79.0)	72.0 (66.0, 77.0)	72.0 (67.0, 77.0)	71.0 (67.0, 76.0)
Men, n (%)	494 (48.4)	141 (74.2)	1624 (54.9)	449 (60.0)	284 (54.0)
Emphysema/COPD, n (%)	85 (8.3)	82 (43.2)	552 (18.7)	119 (15.9)	65 (12.4)
Interstitial pneumonia, n (%)	17 (1.7)	8 (4.2)	95 (3.2)	22 (2.9)	17 (3.2)
BMI <18 kg/m^2^, n (%)	46 (4.5)	13 (6.9)	191 (6.5)	47 (6.3)	27 (5.2)
Pack-years ≥30, n (%)	296 (30.1)	89 (48.4)	1012 (36.0)	266 (38.3)	179 (37.8)
Partial resection (n = 8242)					
N	4446	531	2298	402	565
Median age (IQR) (years)	72.0 (66.0, 78.0)	75.0 (70.0, 79.0)	74.0 (69.0, 80.0)	74.0 (68.0, 79.0)	74.0 (67.0, 79.0)
Men, n (%)	2453 (55.2)	436 (82.1)	1555 (67.7)	295 (73.4)	366 (64.8)
Emphysema/COPD, n (%)	380 (8.6)	180 (33.9)	448 (19.5)	78 (19.4)	80 (14.2)
Interstitial pneumonia, n (%)	159 (3.6)	48 (9.0)	141 (6.1)	29 (7.2)	35 (6.2)
BMI <18 kg/m^2^, n (%)	250 (5.7)	59 (11.3)	156 (6.9)	32 (8.0)	34 (6.1)
Pack-years ≥30, n (%)	1425 (34.4)	302 (63.6)	1006 (47.1)	177 (50.4)	230 (44.6)

IQR: interquartile range; COPD: chronic obstructive pulmonary disease; BMI: body mass index; PGAS: polyglycolic acid sheets

The patterns observed between the segmentectomy and partial resection patients were generally similar to those observed in the lobectomy patients. Patients undergoing partial resection were slightly older and had a higher frequency of IP diagnosis and a smoking history of pack-years ≥30 compared to patients undergoing the other 2 procedures.

### Hospital characteristics and material use

Fewer patients underwent lobectomy, segmentectomy, or partial resection in hospitals with fewer than 200 beds (**Table 3**). Overall, material-use patterns were similar between hospitals with 200–499 beds and those with 500 beds or more across all 3 procedures, with the exception of a segmentectomy, for which the frequencies of the Buttress group or PGAS/Sealant + Hemostatics group were slightly higher in 200–499-bed hospitals. Among patients undergoing partial resection, hospitals with 500 beds or more tended to have a higher proportion of the Stapler-only group compared with 200–499-bed hospitals. The highest proportion of the Buttress group was observed among patients who underwent lobectomy in hospitals with fewer than 200 beds, at 10.0%.

**Table 3:** Material use for preventing prolonged air leakage after lung resection for primary lung cancer by hospital type

Hospital type		Stapler plus				
	Total N	A) Stapler only	B) Buttress	C) PGAS and/or fibrin glue	D) PGAS and/or fibrin glue + others	E) Others
Lobectomy (n = 33094)						
N (%)		7251 (21.9)	743 (2.2)	14329 (43.3)	6228 (18.8)	4543 (13.7)
Hospital scale (no. of beds)						
<200 beds, n (%)	468	101 (21.6)	47 (10.0)	160 (34.2)	87 (18.6)	73 (15.6)
200–499 beds, n (%)	13872	2856 (20.6)	245 (1.8)	6476 (46.7)	2691 (19.4)	1604 (11.6)
500+ beds, n (%)	18754	4294 (22.9)	451 (2.4)	7693 (41)	3450 (18.4)	2866 (15.3)
Cancer treatment facility, n (%)	17764	6363 (35.8)	461 (2.6)	12821 (72.1)	5539 (31.2)	4119 (23.2)
Segmentectomy (n = 5443)						
N (%)		1021 (18.8)	190 (3.5)	2958 (54.3)	748 (13.7)	526 (9.7)
Hospital scale (no. of beds)						
<200 beds, n (%)	36	15 (41.7)	2 (5.6)	14 (38.9)	5 (13.9)	0 (0)
200–499 beds, n (%)	2161	381 (17.6)	95 (4.4)	1112 (51.5)	359 (16.6)	214 (9.9)
500+ beds, n (%)	3246	625 (19.3)	93 (2.9)	1832 (56.4)	384 (11.8)	312 (9.6)
Cancer treatment facility, n (%)	4907	921 (18.8)	129 (2.6)	2684 (54.7)	693 (14.1)	480 (9.8)
Partial resection (n = 8242)						
N (%)		4446 (53.9)	531 (6.4)	2298 (27.9)	402 (4.9)	565 (6.9)
Hospital scale (no. of beds)						
<200 beds, n (%)	243	164 (67.5)	15 (6.2)	54 (22.2)	7 (2.9)	3 (1.2)
200–499 beds, n (%)	3610	1731 (48)	229 (6.3)	1232 (34.1)	203 (5.6)	215 (6)
500+ beds, n (%)	4389	2551 (58.1)	287 (6.5)	1012 (23.1)	192 (4.4)	347 (7.9)
Cancer treatment facility, n (%)	7149	4037 (56.5)	383 (5.4)	1855 (25.9)	351 (4.9)	523 (7.3)

Percentages are calculated with the total of each row as 100%.

PGAS, polyglycolic acid sheets

### Outcomes

Among the patients who underwent lobectomy, the highest incidence of prolonged drainage of 7 days or longer, the primary outcome, was observed in the Buttress group, with 184 out of 743 patients (24.8%), followed by the PGAS/Sealant + Hemostatics group, with 941 out of 6228 patients (15.1%) (**Table 4**). The groups considered to have lower risk exhibited lower incidences of the primary outcome, with 500 out of 7251 patients (6.9%) in the Stapler-only group and 402 out of 4543 patients (8.9%) in the Other materials group. The incidences of reoperation for lung fistula and the use of adhesion therapy were lower but followed the same pattern as the primary outcome, with the Buttress group having the highest incidence, followed by the PGAS/Sealant + Hemostatics group.

**Table 4:** Postoperative outcomes after lung resection by material use category and procedure type

	Stapler plus				
	A) Stapler only	B) Buttress	C) PGAS and/or fibrin glue	D) PGAS and/or fibrin glue + others	E) Others
Lobectomy (n = 33094)					
N	7251	743	14329	6228	4543
Prolonged drainage					
≥7 days, n (%)	500 (6.9)	184 (24.8)	1657 (11.6)	941 (15.1)	402 (8.9)
≥3 days, n (%)	2782 (38.4)	445 (59.9)	7571 (52.8)	3570 (57.3)	2173 (47.8)
Reoperation for lung fistula, n (%)	19 (0.3)	11 (1.5)	105 (0.7)	62 (1.0)	25 (0.6)
Use of the adhesion therapy drug, n (%)	138 (1.9)	48 (6.5)	597 (4.2)	277 (4.5)	138 (3.0)
Segmentectomy (n = 5443)					
N	1021	190	2958	748	526
Prolonged drainage					
≥7 days, n (%)	49 (4.8)	28 (14.7)	195 (6.6)	92 (12.3)	43 (8.2)
≥3 days, n (%)	326 (31.9)	93 (49.0)	1059 (35.8)	387 (51.7)	205 (39.0)
Reoperation for lung fistula, n (%)	4 (0.4)	0 (0.0)	23 (0.8)	5 (0.7)	1 (0.2)
Use of the adhesion therapy drug, n (%)	12 (1.2)	9 (4.7)	67 (2.3)	29 (3.9)	14 (2.7)
Partial resection (n = 8242)					
N	4446	531	2298	402	565
Prolonged drainage					
≥7 days, n (%)	101 (2.3)	45 (8.5)	156 (6.8)	44 (11.0)	30 (5.3)
≥3 days, n (%)	820 (18.4)	172 (32.4)	675 (29.4)	179 (44.5)	169 (29.9)
Reoperation for lung fistula, n (%)	7 (0.2)	3 (0.6)	16 (0.7)	3 (0.8)	4 (0.7)
Use of the adhesion therapy drug, n (%)	45 (1.0)	14 (2.6)	70 (3.1)	23 (5.7)	10 (1.8)

PGAS: polyglycolic acid sheets

The findings in the segmentectomy patients displayed a similar pattern in terms of material-use groups compared to the lobectomy patients, although the incidences of the primary and other outcomes were lower. Among the patients who underwent partial resection, the Buttress group, on the contrary, exhibited a lower incidence of prolonged drainage of 7 days or more, which was 45 out of 531 patients (8.5%), as compared to the PGAS/Sealant + Homostatics group, which was 44 out of 402 patients (11.0%). The pattern was similar with respect to the lower incidences of reoperation for lung fistula and the use of adhesion therapy in the Buttress group (3 out of 531 patients [0.6%] and 14 out of 531 patients [2.6%], respectively) as compared to the PGAS/Sealant + Hemostatics group (3 out of 402 patients [0.8%] and 23 out of 402 patients [5.7%], respectively).

### Postoperative length of stay and hospitalization cost associated with prolonged drainage

The median length of stay after index surgery (25th–75th percentile) among patients without prolonged drainage was similar across the 3 procedures: 8 (6–10) days for lobectomy, 7 (6–10) days for segmentectomy, and 7 (4–9) days for partial resection. Conversely, patients with prolonged drainage experienced longer postoperative lengths of stay: 15 (12–22) days for lobectomy, 15 (12–20) days for segmentectomy, and 15 (11–22) days for partial resection (**Table 5**).

**Table 5:** Length of stay and hospital cost by the presence of prolonged drainage for patients undergoing lung resection for primary lung cancer

	Postoperative drainage period	
	<7 days	≥7 days
Lobectomy		
Cost (thousand JPY)	277 (218–359)	517 (395–840)
Length of stay after surgery (days)	8 (6–10)	15 (12–22)
Segmentectomy		
Cost (thousand JPY)	258 (198–336)	545 (403–859)
Length of stay after surgery (days)	7 (6–10)	15 (12–20)
Partial resection		
Cost (thousand JPY)	227 (153–307)	544 (378–938)
Length of stay after surgery (days)	7 (4–9)	15 (11–22)

*Numbers are median (25th–75th percentiles).

JPY: Japanese yen

**Fig. 2:**
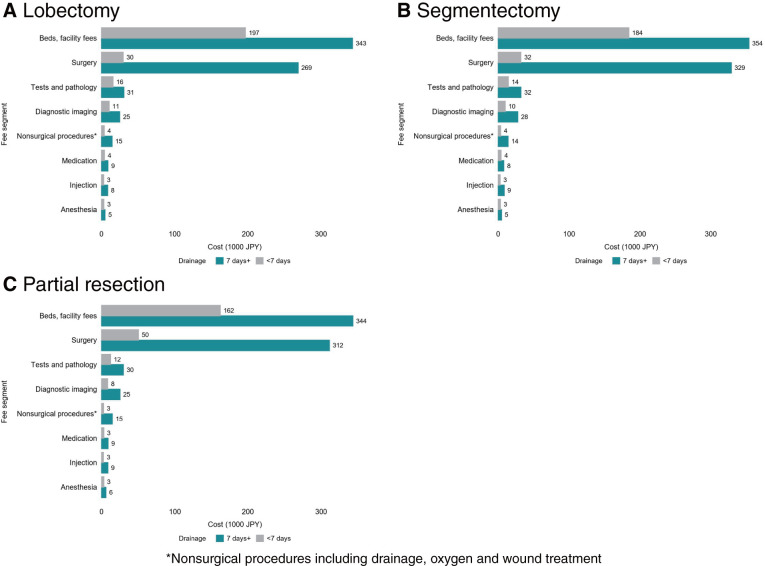
Postoperative hospital cost for patients with and without prolonged drainage after lung resection for primary lung cancer

Regarding in-hospital costs from postoperative day 1 onwards, patients with prolonged drainage incurred higher costs compared to those without prolonged drainage. The median cost for patients with drainage lasting less than 7 days versus 7 days or longer was 277 thousand yen versus 517 thousand yen for lobectomy, 258 thousand yen versus 545 thousand yen for segmentectomy, and 227 thousand yen versus 544 thousand yen for partial resection (**Table 5**). The increased costs primarily stemmed from the extended duration of hospitalization (bed and facility fees) and the expenses associated with additional surgical or interventional procedures (**Fig. 2**).

## Discussion

In an analysis involving 46779 patients who underwent lung resection for primary lung cancer, as identified in an administrative hospital database in Japan, we examined the use of intraoperative materials for the prevention of postoperative lung fistula and assessed the incidence of prolonged drainage and the associated in-hospital costs. We found that PGAS and/or fibrin glue were mostly used in lobectomy and segmentectomy (43.3% and 54.3%, respectively), while in partial resection the Stapler-only group was prevalent. Across procedures, patients who received buttress materials or a combination of materials, including PGAS and/or fibrin glue along with others, exhibited a greater prevalence of preoperative comorbidities such as emphysema/COPD and IP. These groups had higher incidences of prolonged drainage lasting 7 days or longer, which were associated with an increase in in-hospital costs.

Many studies have documented the utilization and efficacy of medical materials in preventing lung fistula after lung resection. Many of these reports are derived from clinical trials and are confined to a limited number of participating institutions.^9–11,17)^ Others stem from retrospective observational studies collected from 1 or more hospitals.^14,18)^ However, no study has reported their usage patterns in routine surgical care using large-scale databases covering hundreds of hospitals.

The findings of our study should be interpreted in the context of routine pre- and intraoperative decision-making. The use of staplers alone is typically selected when the surgeon judges the risk of postoperative air leakage to be low based on pre- and intraoperative findings and lung parenchymal quality. Accordingly, the Stapler-only group in this study should be understood as representing surgeon-selected low-risk cases, in which adjunctive materials were intentionally not used. Similarly, the selection of buttress materials in lobectomy patients appears to reflect surgeon assessment of particularly high risk for postoperative air leakage, and the higher incidence of prolonged drainage observed in this group is most likely attributable to confounding by indication. Buttress materials tend to be used for patients perceived to be at particularly high risk of postoperative air leakage, such as those with severe emphysema, IP, or markedly fragile lung parenchyma. As a result, differences in outcome incidence across material-use groups should be interpreted primarily as reflecting underlying differences in patient risk profiles rather than comparative performance of the materials.

Our results highlight the relatively rare use of buttresses in lung resection surgeries within the study period. In our study, buttress use was strongly associated with male gender, low BMI, smoking history, IP, COPD, and emphysema, suggesting that buttress materials were preferentially used in patients at high risk for pulmonary fistula. Interestingly, while the buttress group had the highest incidence of prolonged drainage in lobectomy and segmentectomy cases, this was lower in partial resections compared to the PGAS/Sealant + Hemostatics group. Partial lung resection may appear to represent a setting in which postoperative air leakage is more closely related to the staple line, and this observation may be consistent with the lower incidence of air leakage observed when buttress materials are used. However, even in this context, postoperative air leakage, particularly among patients with fragile lung parenchyma, may arise from sources other than the staple line, and therefore the findings do not allow definitive conclusions regarding the effectiveness of buttress materials. In addition, differences in postoperative drainage duration among material-use groups in partial resection may also reflect variation in postoperative management practices rather than differences in air leakage itself.

In the present study, the use of staple-line buttresses was less frequent than expected, possibly due to their additional cost influencing surgical decisions despite evidence of efficacy.^18,19)^ Therefore, it is imperative to determine the specific circumstances under which the benefits of staple-line buttresses justify the additional expense. Buttresses may be particularly beneficial in cases with compromised lung parenchyma (e.g., COPD, IP), pleural changes from adhesion or prior treatment, delayed wound healing (e.g., diabetes, immunodeficiency), or substantial lung resections such as deep partial resection or segmentectomy. However, targeted data for these specific situations are presently lacking. Clearly indicating the situations for which staple-line buttresses are most effective will facilitate more judicious patient selection and improved clinical outcomes.

There are some limitations to consider in our study. First, prolonged drainage duration was used as a surrogate indicator of postoperative air leakage. Although extended drainage is commonly associated with persistent air leaks, drainage duration may also be influenced by institutional protocols, surgeon preference, and postoperative management strategies that are unrelated to air leakage itself. As a result, misclassification of postoperative air leakage is possible, and the observed associations should be interpreted with caution. Second, we relied on an administrative database primarily designed for billing purposes. The accuracy of the diagnosis records in such databases is suboptimal^20,21)^ and may underestimate the prevalence of conditions such as emphysema/COPD and IP. Third, the lack of detailed information on patients' pre- and intraoperative clinical conditions limited our ability to establish a causal relationship between material use and clinical outcomes in our study. As a result, our study design is descriptive in nature, focusing on the utilization of materials and the differences observed in outcomes among the cases.

## Conclusion

Prolonged air leakage remains a significant complication after lung resection and is associated with substantial clinical and economic burden. This nationwide descriptive analysis provides an overview of real-world utilization patterns of materials used for air leakage prevention in routine surgical practice. Future studies incorporating detailed clinical and intraoperative information are needed to evaluate optimal material selection in specific patient populations.

## Supplementary Material

Supplemental Table 1 List of medical materials evaluated

Supplemental Table 2 Tumor characteristics by device combination among patients undergoing lung resection surgeries for lung cancer

## Declarations

### Data availability

The data underlying this article were provided by Medical Data Vision Co., Ltd. (MDV) under a data use agreement and cannot be shared by the authors. Researchers interested in accessing the data should contact MDV directly.
