# Supplementary material for: Material Use for Air Leakage Prevention Post-Lung Resection: Insights from Real-World Data in Japan
Source: Ann Thorac Cardiovasc Surg. 2026 Mar 31;32(1):25-00235. doi: 10.5761/atcs.oa.25-00235 (PMC13043235; doi:10.5761/atcs.oa.25-00235)
Supplement: Supplemental Table 1 — List of medical materials evaluated [file atcs-32-1-25-00235-s001.pdf]

Supplemental Table 1. List of medical materials evaluated

Drug/device category	drug/device name
PGAS	Artificial fiber cloth for transubstantiation (for organ loss reinforcement)
Fibrin Grue	Beriplast P Combi-Set Tissue adhesion 0.5mL2Kit
Fibrin Grue	Beriplast P Combi-Set Tissue adhesion 1mL2Kit
Fibrin Grue	Beriplast P Combi-Set Tissue adhesion 3mL2Kit
Fibrin Grue	Beriplast P Combi-Set Tissue adhesion 5mL2Kit
Fibrin Grue	Bolheal Tissue adhesion 0.5mL4Bot
Fibrin Grue	Bolheal Tissue adhesion 1mL4Bot
Fibrin Grue	Bolheal Tissue adhesion 2mL4Bot
Fibrin Grue	Bolheal Tissue adhesion 3mL4Bot
Fibrin Grue	Bolheal Tissue adhesion 5mL4Bot
Buttress	Artificial fiber cloth for transubstantiation (corresponding to automatic suture device)
Other hemostatics	Absorbent local hemostasis materials derived from starch
Other hemostatics	Slightly fibrous collagen
Other hemostatics	Aron Alpha-A (SANKYO) 0.5g
Other hemostatics	Surgicel Absorbable Hemostat 1.3cmx5.1cm
Other hemostatics	Surgicel Absorbable Hemostat 10.2cmx20.3cm
Other hemostatics	Surgicel Absorbable Hemostat 5.1cmx35.6cm
Other hemostatics	Surgicel Absorbable Hemostat 5.1cmx7.6cm
Other hemostatics	Surgicel Absorbable Hemostat CottonType:5.1cmx2.5cm
Other hemostatics	Surgicel Absorbable Hemostat NewKnit:2.5cmx2.5cm
Other hemostatics	Surgicel Absorbable Hemostat NewKnit:2.5cmx8.9cm
Other hemostatics	Surgicel Absorbable Hemostat NewKnit:7.6cmx10.2cm
Other hemostatics	Surgicel Absorbable Hemostat NewKnit:15.2cmx22.9cm
Other hemostatics	Gelfoam 2cmx6cmx0.7cm
Other hemostatics	TachoSil Tissue Sealing Sheet 3.0cmx2.5cm
Other hemostatics	TachoSil Tissue Sealing Sheet 4.8cmx4.8cm
Other hemostatics	TachoSil Tissue Sealing Sheet 9.5cmx4.8cm

Table 2. Tumor characteristics by device combination among patients undergoing lung resection surgeries for lung cancer

	Device combination				
**Lobectomy**	**A) Stapler alone (n=7221)**	**B) Buttress (n=740)**	**C) PGAS and/or Fibline glue (n=14256)**	**D) PGAS and/or Fibline glue + others (n=6194)**	**E) Others (n=4526)**
Tumour site and size (T)					
- T0, n(%)	2 (0.0)	1 (0.1)	28 (0.2)	8 (0.1)	2 (0.0)
- Tis, n(%)	89 (1.2)	5 (0.7)	170 (1.2)	85 (1.4)	58 (1.3)
- T1, n(%)	4169 (57.7)	363 (49.1)	8030 (56.3)	3251 (52.5)	2407 (53.2)
- T2, n(%)	1937 (26.8)	225 (30.4)	3871 (27.2)	1799 (29.0)	1216 (26.9)
- T3, n(%)	401 (5.6)	70 (9.5)	947 (6.6)	442 (7.1)	286 (6.3)
- T4, n(%)	99 (1.4)	21 (2.8)	267 (1.9)	133 (2.2)	74 (1.6)
- NA, n(%)	524 (7.3)	55 (7.4)	943 (6.6)	476 (7.7)	483 (10.7)
Lymph node (N)					
- N0, n(%)	5921 (82.0)	555 (75.0)	11389 (79.9)	4715 (76.1)	3492 (77.2)
- N1, n(%)	430 (6.0)	78 (10.5)	1131 (7.9)	538 (8.7)	326 (7.2)

- N2, n(%)	336 (4.7)	50 (6.8)	766 (5.4)	448 (7.2)	218 (4.8)
- N3, n(%)	7 (0.1)	3 (0.4)	29 (0.2)	13 (0.2)	10 (0.2)
- NA, n(%)	527 (7.3)	54 (7.3)	941 (6.6)	480 (7.8)	480 (10.6)
Metastatic spread (M)					
- M0, n(%)	6600 (91.4)	665 (89.9)	13110 (92.0)	5555 (89.7)	3990 (88.2)
- M1, n(%)	74 (1.0)	21 (2.8)	163 (1.1)	51 (0.8)	40 (0.9)
- NA, n(%)	547 (7.6)	54 (7.3)	983 (6.9)	588 (9.5)	496 (11.0)
**Segmentectomy**	**A) Stapler alone (n=1018)**	**B) Buttress (n=190)**	**C) PGAS and/or Fibline glue (n=2943)**	**D) PGAS and/or Fibline glue + others (n=742)**	**E) Others (n=523)**
Tumour site and size (T)					
- T0, n(%)	1 (0.1)	0 (0.0)	1 (0.0)	1 (0.1)	0 (0.0)
- Tis, n(%)	57 (5.6)	8 (4.2)	220 (7.5)	50 (6.7)	26 (5.0)
- T1, n(%)	749 (73.6)	125 (65.8)	2188 (74.4)	491 (66.2)	381 (72.9)
- T2, n(%)(非適応)	92 (9.0)	25 (13.2)	245 (8.3)	94 (12.7)	50 (9.6)
- T3, n(%)(非適応)	7 (0.7)	9 (4.7)	44 (1.5)	15 (2.0)	6 (1.2)
- T4, n(%)	8 (0.8)	6 (3.2)	16 (0.5)	4 (0.5)	5 (1.0)
- NA, n(%)	104 (10.2)	17 (9.0)	229 (7.8)	87 (11.7)	55 (10.5)

Lymph node (N)					
- N0, n(%)	884 (86.8)	158 (83.2)	2617 (88.9)	622 (83.8)	449 (85.9)
- N1, n(%)	20 (2.0)	6 (3.2)	42 (1.4)	18 (2.4)	8 (1.5)
- N2, n(%)	9 (0.9)	9 (4.7)	39 (1.3)	10 (1.4)	5 (1.0)
- N3, n(%)	1 (0.1)	0 (0.0)	7 (0.2)	0 (0.0)	0 (0.0)
- NA, n(%)	104 (10.2)	17 (9.0)	238 (8.1)	92 (12.4)	61 (11.7)
Metastatic spread (M)					
- M0, n(%)	901 (88.5)	167 (87.9)	2683 (91.2)	643 (86.7)	461 (88.2)
- M1, n(%)	17 (1.7)	4 (2.1)	33 (1.1)	7 (0.9)	6 (1.2)
- NA, n(%)	100 (9.8)	19 (10.0)	227 (7.7)	92 (12.4)	56 (10.7)

**Partial Resection**	**A) Stapler alone (n=4434)**	**B) Buttress (n=525)**	**C) PGAS and/or Fibline glue (n=2286)**	**D) PGAS and/or Fibline glue + others (n=400)**	**E) Others (n=562)**
Tumour site and size (T)					
- T0, n(%)	5 (0.1)	0 (0.0)	3 (0.1)	1 (0.3)	2 (0.4)
- Tis, n(%)	364 (8.2)	6 (1.1)	113 (4.9)	13 (3.3)	44 (7.8)
- T1, n(%)	2574 (58.1)	313 (59.6)	1379 (60.3)	213 (53.3)	287 (51.1)
- T2, n(%)	331 (7.5)	98 (18.7)	244 (10.7)	61 (15.3)	64 (11.4)

- T3, n(%)	76 (1.7)	20 (3.8)	61 (2.7)	15 (3.8)	15 (2.7)
- T4, n(%)	67 (1.5)	10 (1.9)	28 (1.2)	11 (2.8)	15 (2.7)
- NA, n(%)	1017 (22.9)	78 (14.9)	458 (20.0)	86 (21.5)	135 (24.0)
Lymph node (N)					
- N0, n(%)	3100 (69.9)	389 (74.1)	1596 (69.8)	258 (64.5)	361 (64.2)
- N1, n(%)	72 (1.6)	11 (2.1)	55 (2.4)	22 (5.5)	12 (2.1)
- N2, n(%)	98 (2.2)	20 (3.8)	68 (3.0)	15 (3.8)	14 (2.5)
- N3, n(%)	29 (0.7)	5 (1.0)	23 (1.0)	4 (1.0)	10 (1.8)
- NA, n(%)	1135 (25.6)	100 (19.1)	544 (23.8)	101 (25.3)	165 (29.4)
Metastatic spread (M)					
- M0, n(%)	3152 (71.1)	412 (78.5)	1702 (74.5)	289 (72.3)	389 (69.2)
- M1, n(%)	248 (5.6)	30 (5.7)	108 (4.7)	20 (5.0)	22 (3.9)
- NA, n(%)	1034 (23.3)	83 (15.8)	476 (20.8)	91 (22.8)	151 (26.9)
